# The Role of Vertical and Horizontal Transfer in the Evolutionary Dynamics of *PIF*-Like Transposable Elements in Triticeae

**DOI:** 10.1371/journal.pone.0137648

**Published:** 2015-09-10

**Authors:** Dragomira N. Markova, Roberta J. Mason-Gamer

**Affiliations:** Department of Biological Sciences, University of Illinois at Chicago, Chicago, IL, United States of America; Chinese Academy of Sciences, CHINA

## Abstract

*PIF*-like transposable elements are members of the *PIF*/Harbinger superfamily of DNA transposons found in the genomes of many plants, animals, and fungi. The evolution of the gene that encodes the transposase responsible for mobilizing *PIF*-like elements has been studied in both plants and animals, but the elements' history in flowering plants remains poorly known. In this work, we describe the phylogenetic distribution and evolution of *PIF*-like elements in the genomes of 21 diploid species from the wheat tribe, Triticeae, and we present the first convincing evidence of horizontal transfer of *PIF* elements in plant genomes. A phylogenetic analysis of 240 *PIF* sequences based on the conserved region of the transposase domain revealed at least four main transposase lineages. Their complex evolutionary history can be best explained by a combination of vertical transmission with differential evolutionary success among lineages, and occasional horizontal transfer between phylogenetically distant Triticeae genera. In addition, we identified 127 potentially functional transposase sequences indicating possible recent activity of *PIF*.

## Introduction

Transposable elements (TEs) constitute a significant fraction of plant genomes, accounting for 10–12% of the *Arabidopsis thaliana* genome, and 70–80% of maize, barley, and wheat genomes [1–7]. Their ability to move to new genomic locations results in mutational activity and can alter the structure and the function of individual genes [8, 9]. Therefore, studies of TEs and their impact on host genomes are relevant to understanding genome-wide evolution.

Eukaryotic TEs have been classified into two broad classes according to their mechanism of transposition. Class I TEs, or retroelements, transpose through an element-encoded mRNA intermediate that is reverse-transcribed; the DNA element itself is not mobilized. Class II TEs, or DNA elements, transpose via a double-stranded DNA intermediate through a “cut and paste” mechanism whereby the element is excised and reinserted elsewhere in the host genome [10]. These elements usually have terminal inverted repeats (TIRs) whose size and sequence are characteristic of the family or superfamily to which they belong. Class II elements can be further divided into two group: autonomous elements encode a transposase gene that catalyzes DNA cleavage and transposition; non-autonomous elements do not encode a functional transposase, but can still be transactivated by a transposase coded by a related autonomous element located elsewhere in the genome [11, 12]. All Class II TE superfamilies contain both autonomous and non-autonomous elements. A TE superfamily is defined primarily by sequence similarity of the transposase, the terminal nucleotides of the TIRs, and the length of the target site duplications (TSDs) generated by the transposase upon inserting the element [13]. Seventeen superfamilies of eukaryotic Class II TEs have been proposed so far in eukaryotes [14], with only five found in plant genomes (CACTA, *Mutator*, *PIF/*Harbinger, *h*AT, and *Tc*1*/mariner*) [14, 15].

In this work, we examined the distribution and evolution of an actively transposing family of Class II elements called *P* instability factor (*PIF*) in the genomes of 21 diploid species from the wheat tribe, Triticeae. The Triticeae comprises approximately 30 genera and 300–400 species [16], including some of the world's most economically important crops such as wheat, barley, and rye. The wheat tribe also serves as an interesting model of TE evolution, because TEs account for 70–80% of its species' genomes [2, 3, 5–7].

**Fig 1:**
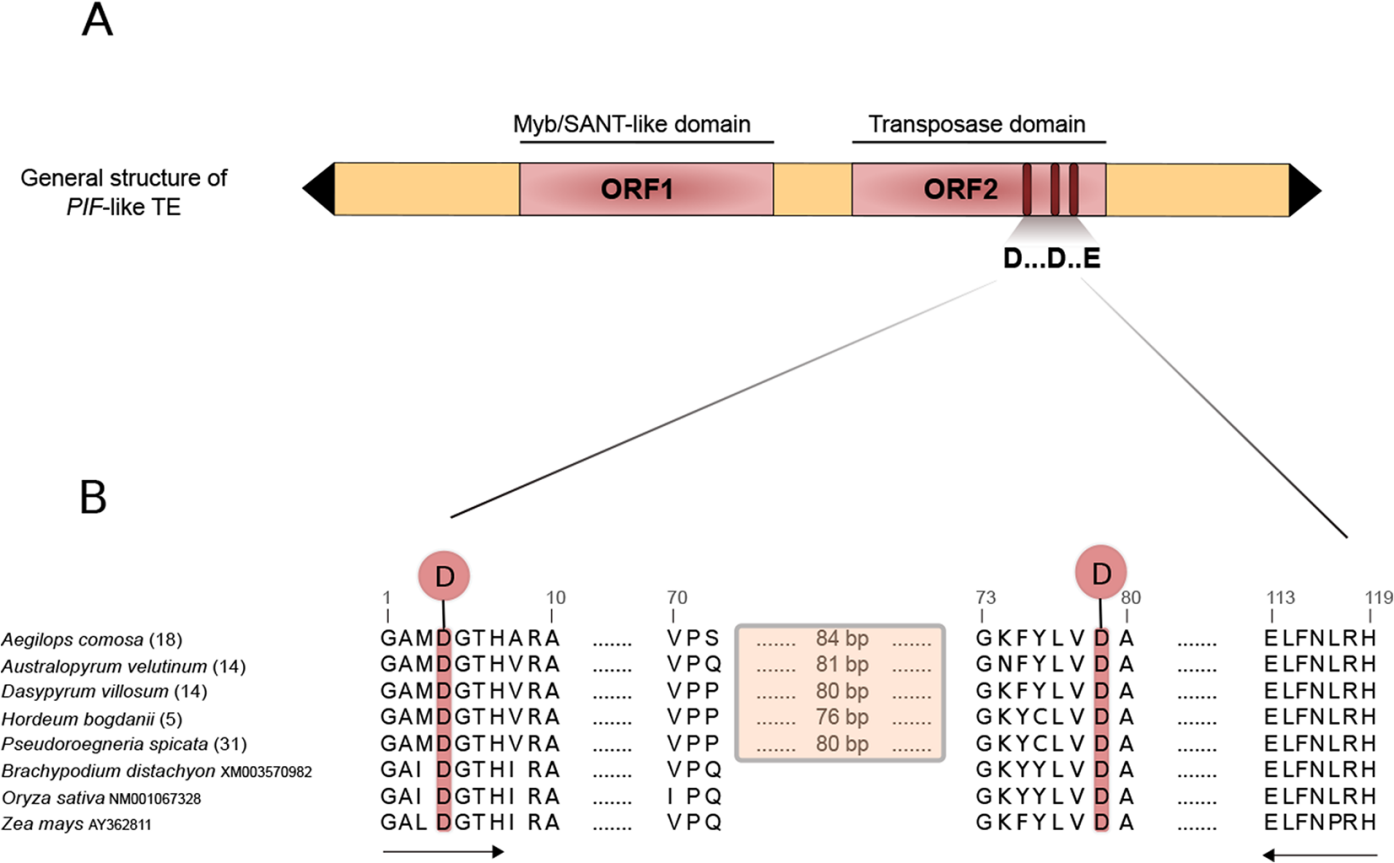
General structure of *PIF*-like elements. A) General structure of a *PIF*-like element with the corresponding ORFs. ORF2 contains a “DDE” motif, a signature consisting of three conserved amino acids. The black triangles represent TIRs and the pink rectangles represent ORFs. B) Comparison of the conserved transposase domain with the corresponding “DD” portion of the conserved “DDE” motif from *Brachypodium*, *Oryza*, and *Zea* (obtained from GenBank), and five representative Triticeae species (generated in this study). The shaded rectangle indicates an intron. Locations of ORF2 amplification primers are indicated by bold arrows. The number in parentheses following each Triticeae taxon name designates a specific cloned sequence from within that individual; these numbers are consistent among Figs 1–3.

*PIF*-like elements belong to the *PIF*/IS5 or *PIF*/Harbinger superfamily of transposons [17]. They were first discovered in the maize genome as six independent insertions into exactly the same site in intron 2 of the R gene [18], and they have since been detected in the genomes of many flowering plants, animals, and fungi [17, 19–21]. Most *PIF* elements are approximately 4–6 kb long [17, 19, 20] and contain two open reading frames (ORFs), one encoding a transposase (ORF2), and one whose function is still unknown (ORF1) (Fig 1A), though it is thought to be involved in DNA binding activity and protein-protein interactions [17, 22–24]. The transposase contains a “DD(34/37)E” motif, a signature consisting of an amino acid triad (Fig 1A) identified in the transposases of most DNA transposon superfamilies [14, 25, 26]. The “DDE” motif consists of two aspartic acid (D) residues and a glutamic acid (E) residue interspersed within a relatively well conserved domain of amino acids, and it has helped to establish the evolutionary relationships among *PIF* elements [17, 20, 21]. In some *PIF* elements the transposase gene is interrupted by between one and three insertions previously characterized as introns [17, 21, 27]. Other characteristics of this superfamily include 14–25 bp TIRs, and the generation of TTA/TAA TSDs upon insertion.

We investigated the evolutionary history of *PIF*-like TEs in Triticeae through a phylogenetic analysis of 240 *PIF* sequences based on a 360-bp region of the conserved transposase gene (Fig 1B). Our results revealed complex patterns that can be best explained by ancestral polymorphism, followed by differential evolutionary success among transposase lineages, and by occasional horizontal transfer (HT) events between genera. In addition, we identified 127 transpose sequences with no frame shifting indels or stop codons, most revealing a signal of purifying selection, indicating that there are probably numerous distinct functional transposase copies.

## Materials and Methods

### Plant material

A total of 22 accessions representing 21 species from 16 Triticeae genera were selected for this study (Table 1). In order to avoid the potentially confounding phylogenetic effects of auto- and allopolyploidy, only diploid taxa were chosen. Nearly all accessions were obtained from the USDA as seed, and have associated chromosome counts. The DNA was extracted for previous phylogenetic studies from fresh or dried leaf material, using a CTAB-based method [28]. We included multiple accessions from *Pseudoroegneria* (sometimes called "wheatgrass") and *Hordeum* (wild barley), the parental genome donors of North American allotetraploid *Elymus* species [29–34], because these will be applicable to future studies of *PIF*-like transposon dynamics in *Elymus* allopolyploid genomes.

**Table 1:** Triticeae samples included in *PIF* analyses.

Species name	Sample source and reference number	GenBank accession
*Aegilops comosa* Sibth. & Smith	USDA/G602	
*Agropyron cristatum* (L.) Gaertn.	USDA/PI 279802	
*Australopyrum velutinum* (Nees) B.K.Simon	USDA/D 2873–2878	
*Crithopsis delileana* (Schult.) Roshev.	USDA/H 5562	
*Dasypyrum villosum* (L.) P.Candargy	USDA/D 2990	
*Eremopyrum bonaepartis* (Spreng.) Nevski	USDA/PI 227344	
*Henrardia persica* (Boiss.) C.E.Hubb	USDA/H 5556	
*Heteranthelium piliferum* (Banks & Sol.) Hochst.	USDA/PI 402352	
*Hordeum bogdanii* Wilensky	USDA/PI 531762	
*Hordeum chilense* Roem. & Schult.	USDA/PI 531781	
*Peridictyon sanctum* (Janka) Seberg, Fred., & Baden	USDA/KJ 248	
*Psathyrostachys fragilis* (Boiss.) Nevski	USDA/PI 343192	
*Psathyrostachys juncea* (Fisch.) Nevski	USDA/PI 206684	
*Pseudoroegneria libanotica* (Hack.) D.R.Dewey	USDA/PI 228391	
*Pseudoroegneria spicata* (Pursh) Á.Löve	USDA/D 2844	
*Pseudoroegneria tauri* (Boiss. & Balansa) Á.Löve	USDA/PI 401319	
*Secale montanum* Guss.	USDA/T 36554	
*Taeniatherum caput-medusae* (L.) Nevski	USDA/PI 208075	
*Taeniatherum caput-medusae*	USDA/PI 283240	
*Thinopyrum bessarabicum* (Săvul. & Rayss) Á.Löve	USDA/PI 531711	
*Triticum monococcum* L.	USDA/PI 221413	
*Triticum urartu* Tumanian ex Gandilyan	Morrison s.n.	
*Brachypodium distachyon* (L.) P.Beauv.		XM003570982
*Oryza sativa* L.		NM001067328
*Zea mays* L.		AY362811

### Amplification of the *PIF* transposase

We targeted the conserved region of the transposase, which corresponds to approximately 440 bp in the majority of our samples, including an 80 bp intron. This represents about 35% of the *PIF* transposase gene, which varies in length from 1176 to 1296 base pairs (bp) [17]. Based on bamboo-specific PCR primers for the amplification of a portion of the transposase gene [21], we designed Triticeae-specific degenerate PCR primers (*PIF*-for: GGAGCHWTNGATGGYACWCAC, *PIF*-rev: TGCCKAAGRTTRAAYARYTC) (Fig 1B). These primers are anchored in two highly conserved amino acid residue motifs (GAMDGTH and ELFNPRH respectively) flanking the DD portion of the “DDE” domain of the transposase gene (Fig 1B). Amplifications were carried out in a 10-μl reaction mixture containing 50 ng of genomic DNA, 10x PCR buffer, 0.2 mmol/L primer pair, 0.5 units of Taq polymerase (Sigma), 0.2 mmol/L of each dNTP, and 1.5 mmol/L MgCl_2_. The PCR amplification conditions were as follows: 5 min of DNA denaturation at 95°C; 35 cycles of 30 s at 95°C, 45 s at 57°C and 60 s at 72°C; and a 10 min final extension at 72°C.

### Cloning, sequencing, and sequence alignment

All PCR products were cloned prior to sequencing. Three PCR reactions were run for each cloning reaction to counter the potential effects of PCR drift [35]. PCR products from replicated reactions were isolated on 1% agarose gels, combined, and purified on columns (Qiagen). Cleaned products were cloned into pGEM-T Easy vectors (Promega) and transformed into *E*. *coli* JM109 competent cells (Promega) according to the manufacturer’s instructions, except that all reactions were halved. Colonies containing the insert were subjected to PCR reactions as described above. The resulting fragments were cleaned with 0.2 μl exonuclease I and 0.4 μl shrimp alkaline phosphatase, and sequenced in both directions with the PCR primers. All cloning steps described above were repeated at least three times for each sample. Sequencing was performed on an ABI 377 automated sequencer (Applied Biosystems). A minimum of 24 clones per sample were sequenced, including no more than 8 clones per cloning reaction, in order to evaluate transposase diversity within individuals.

The coding sequences of *PIF*-like transposases were aligned using CLUSTALW [36] with default parameters, and then manually adjusted in MacClade 4.08 [37]. The first alignment consisted only of sequences with no frame shifting indels or stop codons, so the reading frame indicated in [21] was used to aid in the alignment. The remaining sequences were then added to the initial alignment and all insertions and deletions were adjusted accordingly. Duplicate sequences from within individuals were excluded from the data set, as were those that differed by 1 bp based on the consensus sequence, under the assumption that they represent potential Taq errors. All sequences were deposited in the NCBI GenBank database (accession numbers KT024997-KT025236).

### Phylogenetic analyses

Phylogenetic analyses of the conserved transposase domain in Triticeae were performed on a region of approximately 360 bp coding sequence; the intron exhibited considerable sequence variation, and was thus excluded from subsequent analyses because of alignment ambiguity. We analyzed two data sets. The first consisted of 127 potentially functional transposase sequences, i.e., those with no frame shifting mutations or stop codons (reduced data set). The assumption that these sequences are potentially functional is very preliminary because we do not have the entire gene sequence, and because we would not have detected missense mutations leading to loss of function. The second data set contained all 240 transposase sequences that we generated (complete data set), including those with stop codons or frame shifting indels (and thus do not appear to code for a functional protein). Pairwise sequence distances were calculated from the multiple alignment using PAUP* 4.0b10 [38]. Phylogenies were estimated using maximum parsimony (MP) and maximum likelihood (ML). Parsimony analyses were conducted with PAUP* v.4.0b10 [38] with heuristic searches. The parsimony bootstrap (BS) method, with 1000 replicates with heuristic search, was used to estimate the robustness of the clades [39] (tree not shown). For the ML analyses, the appropriate models of sequence evolution were determined using jModelTest [40–42] and the corrected Akaike information criterion [43]. The selected models, GTR+Γ and GTR+Γ+I for the reduced and the complete data set, respectively, were used for ML analyses in GARLI v.0.95 [44]. Following the recommendations of the author, multiple (fifty) analyses with random starting tree topologies were performed for each data set. Runs were set for an unlimited number of generations, and automatic termination following 10,000 generations without a significant change in topology. Bootstrap support for each tree was estimated based on 100 ML bootstrap replicates with the same options used to generate the ML tree.

### Sequence variability and evolution

The synonymous (*d*S) and non-synonymous (*d*N) substitution rates were estimated and compared (*d*N/*d*S) for the 127 transposase sequences with no frame shifting indels or stop codons, to test for purifying or adaptive selection. Accordingly, *d*N/*d*S ratios near 1 reflect neutral sequence evolution, while ratios less than 1 indicate negative or purifying selection due to selective constraints, and values greater than 1 indicate positive selection, suggesting adaptive evolution. After removing one of a pair of identical sequences (*Thinopyrum bessarabicum* 8, which is identical to *Pseudoroegneria libanotica* 14) to avoid division by zero, *d*N/*d*S ratios and codon based Z-tests were calculated through a modified [45] method with a Jukes-Cantor correction, as implemented in the MEGA5.0 software [46].

## Results

### Isolation and characterization of *PIF*-derived transposases

PCR amplifications yielded a single band of approximately 440 bp for all samples, representing 360 bp of coding sequence with a 72–88 bp intron. *Psathyrostachys juncea* and *Hordeum bogdanii* had two bands of 440 bp and 360 bp respectively, with the shorter fragments lacking the intron. Sequence comparison of Triticeae representatives to other members of the Poaceae (Fig 1B) revealed the highly conserved structure of the sequenced portion of the transposase, with the first D of the conserved “DDE” motif at position 4 and the second D at position 79 of the consensus amino acid sequence, each surrounded by relatively well conserved amino acid blocks. The intron begins six residues upstream of the second D (Fig 1B).

**Fig 2:**
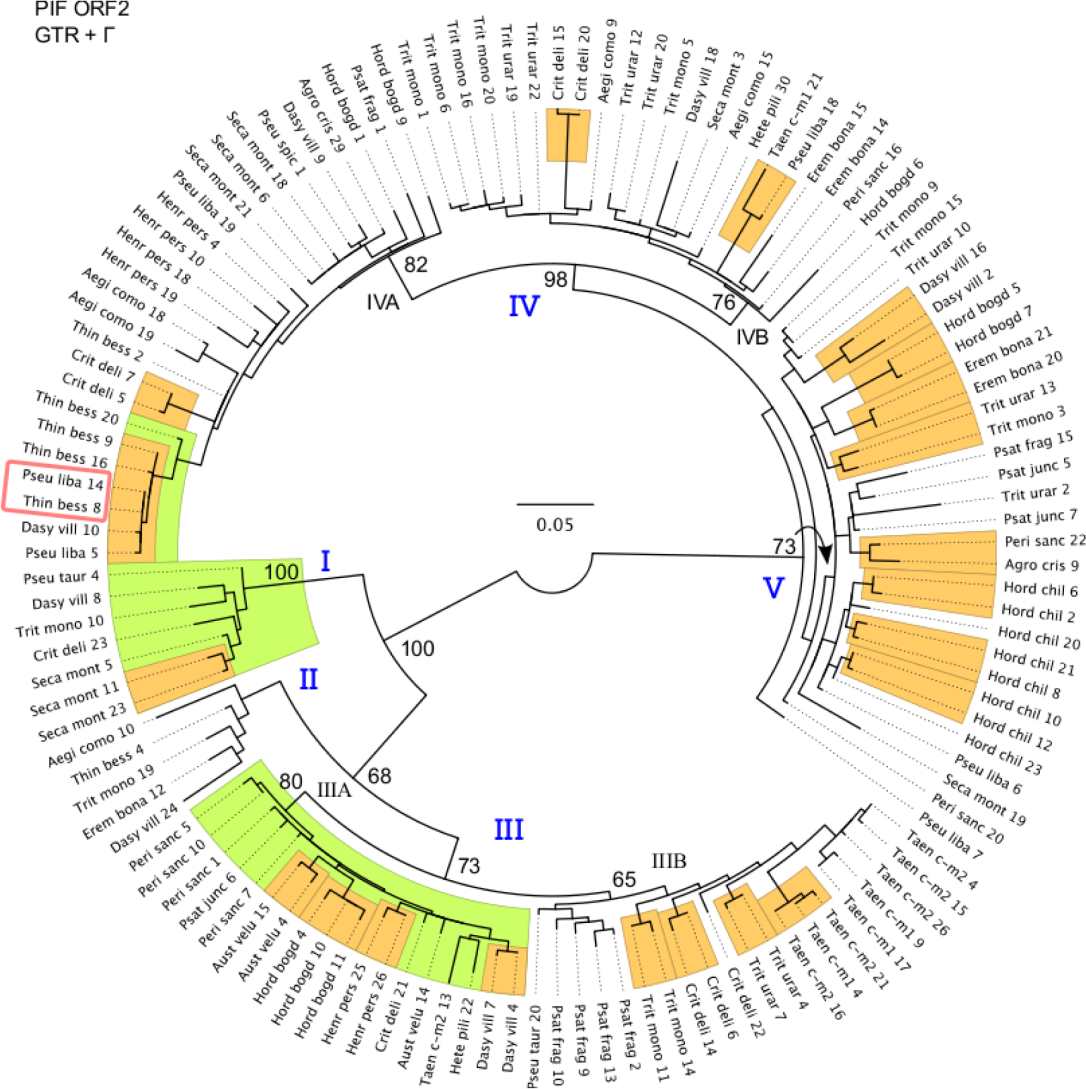
Phylogenetic analysis of the conserved transposase domain of 127 *PIF*-like transposase sequences. The reduced data set includes potentially functional coding sequences with no indels or stop codons. The best ML tree was selected from 50 GARLI analyses under the GTR+Γ model of sequence evolution. Yellow and green rectangles show clades with BS above 80%; numbers indicate BS values >50% on deeper nodes. The red rectangle indicates a pair of identical transposase sequences from distinct genera. Taxon labels combine the first four letters of the genus and species names. Numbers following taxon names distinguish individuals within species, and numbers in parentheses designate specific cloned sequences from within individuals and are consistent among Figs 1–3.

Of the approximately 500 sequenced fragments, 240 were unique (complete data set), indicating the presence of multiple distinct transposases in each species. All sequences contained the “DD” portion of the “DDE” motif. Significant heterogeneity was found within individuals, and each taxon was represented by a set of nonredundant clones. Of the 240 nonredundant sequences, 113 had frame shifting indels or stop codons, and thus are probably not functional. The remaining 127 fragments (reduced data set) were aligned and used to generate the phylogenetic tree in Fig 2, on which they fall into multiple evolutionary lineages, indicating that multiple transposases have the potential to function simultaneously within a genome.

### Sequence variation of *PIF*-like transposase sequences

*PIF* transposase sequences in Triticeae showed 85.69–100% nucleotide sequence identity for the reduced data set (127 fragments) and 72.5–100% sequence identity for the complete data set (all 240 *PIF* sequences generated; data not shown), with the highest level of divergence found between sequences from *Eremopyrum bonaepartis* and *Crithopsis delileana*. Comparison with a typical *PIF* element from corn (*Zea mays*; AY362811) revealed 71.5–84.5% sequence similarity for the complete data set.

**Fig 3:**
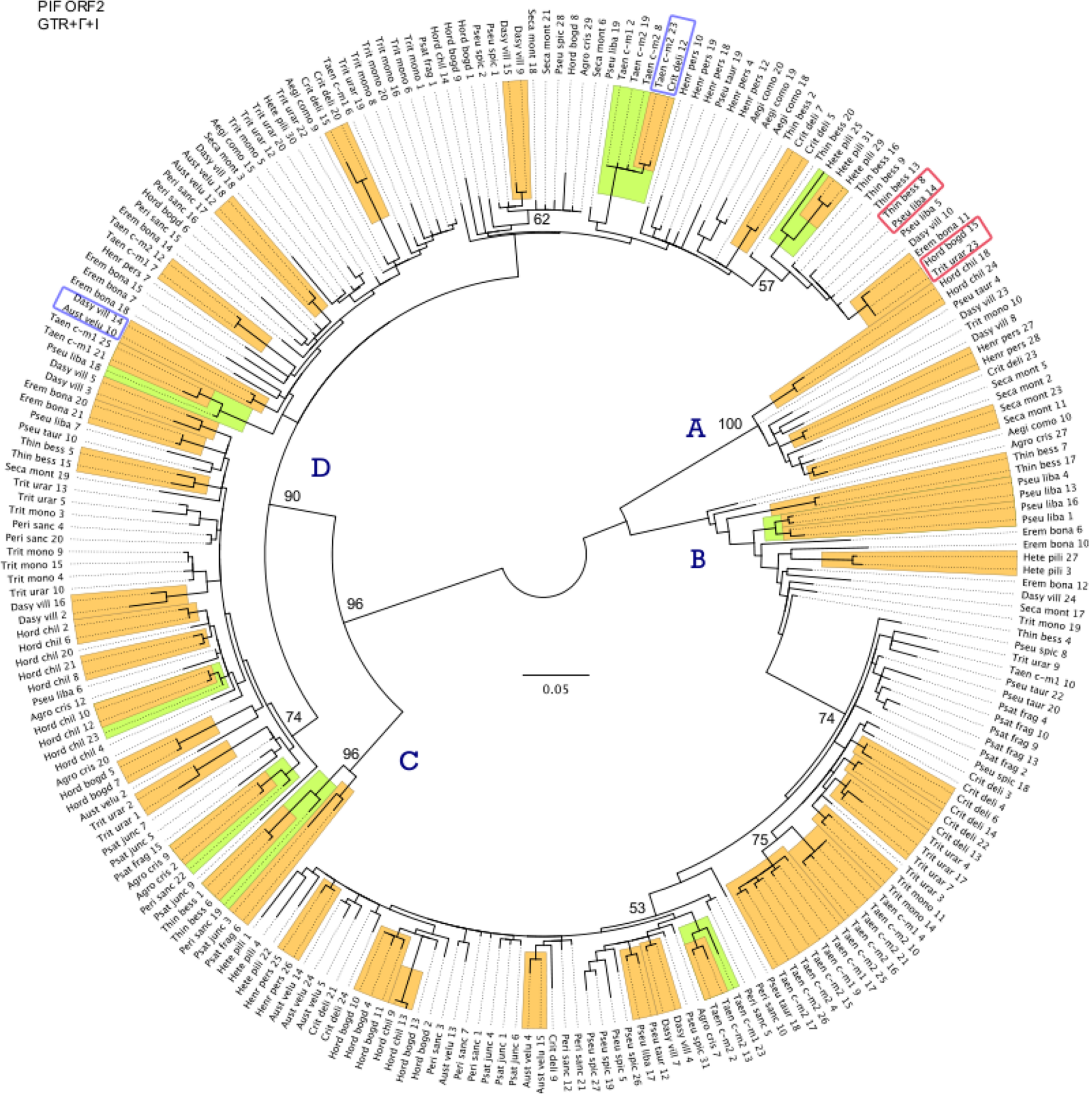
Phylogenetic analysis of 240 *PIF*-like transposase sequences from Triticeae. The complete data set includes transposase sequences with indels or stop codons. The best ML tree was selected from 50 GARLI analyses under the GTR+Γ+I model of sequence evolution. Orange and green rectangles show clades with BS above 80%; numbers indicate BS values >50% on deeper nodes. Red rectangles indicate identical transposase sequences from distinct genera. Blue rectangles indicate highly similar transposase sequences from distinct genera. Taxon labels combine the first four letters of the genus and species names. Numbers following names distinguish cloned sequences from within individuals and are consistent among Figs 1–3.

Transposase fragments with identical coding sequences were shared across genus boundaries in two cases (marked with red rectangles on Fig 3): *Pseudoroegneria tauri* 14 was identical to *T*. *bessarabicum* 8 (94.45% sequence identity over the 73-bp intron), and *H*. *bogdanii* 15 shared 100% sequence identity to *Triticum urartu* 23 (98.61% sequence identity over the 71-bp intron).

### Phylogeny of *PIF*-like transposases

Maximum parsimony topologies (not shown) were in general accordance with the ML topologies; however, there was higher resolution and more robust support in the ML trees. In previous studies of *PIF*-like evolution in bamboo, a sequence from a fungus (*Filobasidiella neoformans*; AC068564) was used as an outgroup [17, 21]. We decided that this choice was not appropriate for our data given the high sequence divergence from our ingroup (51.8–66.3%), and thus the possibility of increased homoplasy. We did not have a clearly better outgroup, so we used the mid-point rooting method for all of the phylogenetic trees [47]. We could not use *PIF* sequences from grasses outside the wheat tribe to root the tree, because the root of the *PIF* element tree appears to predate the origin of the wheat tribe (i.e., some *PIF* elements from within the Triticeae are more closely related to elements from outside of the wheat tribe than they are to other elements within the wheat tribe; see also [17, 21]).

### Phylogeny of reduced data set

The first data set contained 127 *PIF*-like transposase sequences with no frame shifting indels or stop codons. In the best topology (Fig 2;-lnL = 4415.5775), many clades received strong bootstrap support. Triticeae transposase sequences form five main groups (I–V in Fig 2); some subgroups within groups were labeled with letters (A, B, etc.).

Group I received strong support (100% BS) and consisted of sequences from *P*. *tauri*, *Dasypyrum villosum*, *Triticum monococcum*, *C*. *delileana*, and *Secale montanum*. Group II was weakly supported (BS < 50%) and included sequences from *Aegilops comosa*, *T*. *bessarabicum*, *T*. *monococcum*, *E*. *bonaepartis*, and *D*. *villosum*. Within Group III (73% BS), two subgroups (IIIA and IIIB) were identified with 80% and 65% branch support, respectively. Group III was represented by sequences from all except eight samples (*Pseudoroegneria libanotica*, *T*. *bessarabicum*, *S*. *montanum*, *Hordeum chilense*, *E*. *bonaepartis*, *Pseudoroegneria spicata*, *Agropyron cristatum*, and *A*. *comosa*).

*Pseudoroegneria libanotica* 7 was sister to group IV+V. Group IV (98% BS) was the largest and by far the most complex, and was further subdivided into two subgroups (IVA and IVB; 82% and 76% BS, respectively; Fig 2). Group IV contained sequences from all but six individuals: *Taeniatherum caput-medusae* 2, *P*. *tauri*, *Australopyrum velutinum*, *H*. *chilense*, *Heteranthelium piliferum*, and *Psathyrostachys fragilis*. Within this cluster, *T*. *bessarabicum* 8 was identical to *P*. *libanotica* 14 (marked with rectangles on Fig 2), possibly reflecting introgression among genera. Group V (73% BS) included sequences from numerous Triticeae genera, with one cluster mostly represented by *H*. *chilense* fragments. *Dasypyrum villosum* and *T*. *monococcum* were distributed throughout the tree, in all evolutionary groups.

### Phylogeny of complete data set

The second data set included 240 non-redundant *PIF*-like transposase sequences from all 22 accessions (Table 1; complete data set), including the presumably non-functional copies with frame shifting indels and/or stop codons. The best topology (-lnL = 8235.58335; Fig 3) revealed four main groups of *PIF* transposases in Triticeae (A–D in Fig 3). Group D (90% BS) was the largest and by far the most complex, and was further subdivided into three subgroups. Group D contained sequences from all 22 samples. Group B (BS < 50%) was the second most diverse and was represented by sequences from all samples except *A*. *comosa*. Groups A and C (100% and 96% BS, respectively) included sequences from fewer taxa (Fig 3). Clusters with a limited number of representative species probably represent a combination of the loss of some *PIF* lineages through stochastic events and natural selection, and random sampling artifacts.

Two pairs of transposases from distinct genera exhibited identical coding regions (marked with red rectangles on Fig 3): *T*. *bessarabicum* 8 was identical to *P*. *libanotica* 14; and *H*. *bogdanii* 15 was identical to *T*. *urartu* 23.

### Detecting selection in the transposase domain

A functional gene, or one that was recently functional, is expected to show a signature of strong selective pressure. *d*N/*d*S comparisons of 126 transposases with no frame-shifting and/or stop codons showed a dominant signal of purifying selection (*d*N/*d*S < 1) with average *d*N/*d*S ratio of 0.203 (S1 and S2 Tables). Although the Z-test P-values were above the cutoff of 0.05 for some sequence comparisons (in red in S3 Table), most pairwise comparisons rejected the null hypothesis of neutral evolution. These results indicate that purifying selection dominated the evolution of this sample of transposase sequences, consistent with characteristics of a functional gene.

## Discussion

Since their original discovery in *Zea mays* [18], *PIF*-like transposases have been found in the genomes of many plants, animals, and fungi [17, 19–21]. The ubiquity of *PIF*-like elements suggests an early origin of the group. In plants, *PIF* elements are ancient, widespread, diverse, and abundant, and they probably evolve independently from their hosts [17, 19–21]. Here we report the first comprehensive phylogenetic analysis of *PIF*-like TEs from the wheat tribe. We have isolated, cloned, and sequenced a portion of the transposase domain of *PIF*-like TEs in 22 Triticeae samples in order to (1) examine the sequence variability of this region within the wheat tribe; (2) determine whether the existing *PIF* transposase lineages evolved prior the origin of the wheat tribe; and (3) identify possible introgression between species.

### Evolution of *PIF*-like elements in Triticeae

The evolution of *PIF*-like elements within Triticeae is complex. Phylogenetic analyses of both the reduced and the complete data sets (Figs 2 and 3) reveal that multiple distinct transposase lineages coexist within genomes. The existence of such divergent TE sequences in a single host genome has already been reported for other elements such as Pong [48], *mariner* [49], *P* [50], and *Minos* [51], and is best explained by ancient episodes of element diversification in early ancestors, followed by vertical propagation. Both of the *PIF* topologies (Figs 2 and 3) are consistent with the vertical transmission of diverse ancestral transposase lineages. However, cases where identical transposase sequences were detected in different genera suggest that occasional HT events also played an important role in the complex pattern of distribution of transposases in the wheat tribe.

### Vertical transmission of *PIF*-like transposases

Phylogenetic analyses (Figs 2 and 3) show vertical transmission of multiple diverse ancestral transposase lineages, with some lineages experiencing differential evolutionary success. For example, the broad representation of taxa in Groups B and D (Fig 3) suggests extensive diversification of these transposases in a common ancestor prior the origin of the wheat tribe (13–25 mya; [52]), followed by vertical propagation and further diversification of elements. Group B is only missing from *A*. *comosa*, which may be due to stochastic events, natural selection, and/or a sampling artifact where we did not detect all of the sequences.

In contrast, Groups A and C are represented in far fewer individuals (Fig 3). While this could be due to differential amplification and retention of elements, Group A is found in species that are broadly representative of Triticeae phylogenetic diversity, including presumably basal branches of the wheat tribe such as *Hordeum* and *Psathyrostachys* [34, 53]. This indicates that Group A was probably already present at the time of Triticeae radiation, and was lost from some of the descendent genomes. Variation in the success of other DNA transposons among Triticeae species has already been reported. For example, the CACTA DNA element *Jorge* accounts for only 0.03% of the *Hordeum vulgare* and *H*. *spontaneum* genomes, in contrast to 4.93% of *Aegilops tauschii* genome [54]. The recently discovered transposon-like gene *Revolver* [55] is found in extremely high copy numbers in the Triticeae species *D*. *villosum* and *Secale* sp. (~20,000), and in *T*. *monococcum* (~10,000), while there are virtually no copies in the bread wheat genome (*Triticum aestivum*) [53]. All transposable elements share the basic ability to amplify their copy number within a genome. However, due to their potential impact on the structure and function on host genes, TE content within a genome is governed by a combination of transposition and sequence removal via genetic drift and natural selection. Thus, it is expected that some copies will be lost or retained within a genome, and that the retained copies have the potential to evolve differently in different genomes.

### Horizontal transfer of *PIF*-like transposases

Past or ongoing HT events also appear to have contributed to the distribution of *PIF*-like elements in Triticeae. This hypothesis is supported by the presence of nearly-identical transposase fragments shared among genera in two cases (marked with red rectangles in Fig 3). These genera diverged about 13–25 mya [52], so it is very unlikely that their highly conserved transposase sequences diverged at the same time as the hosts and maintained near identity, even under strong selective constraints, because selection will not prevent the accumulation of synonymous changes through time. Moreover, one of the two pairs of sequences (*H*. *bogdanii* 15 and *T*. *urartu* 23) has at least one stop codon, and is thus probably non-functional and therefore not under selective constraints. In addition to these two cases, we detected at least two more pairs of sequences with extremely high levels of sequence similarity in their coding regions: *C*. *delileana* 12 and *T*. *caput-medusae*2 23; and *D*. *villosum* 14 and *A*. *velutinum* 10 (marked with blue rectangles in Fig 3), which further suggest gene exchange among Triticeae genera.

High sequence similarity among elements in divergent taxa is one of several criteria (e.g.,[56]) that have been used to infer transposon HT. Documented cases include other Class II elements, such as *mariner* [57, 58], *P* elements [59, 60], and *PIF*-like elements in *Drosophila* [19], as well as Class I LTR retrotransposons [56]. Although purifying selection or convergent evolution can also result in sequence similarity among taxa, neither of these processes would prevent the accumulation of neutral changes through millions of years of independent evolution in their respective genera. We have taken a very conservative approach to inferring HT rather than selection by highlighting only those cases where the coding sequences shared between genera are fully identical. The lack of any synonymous differences indicates a very recent common ancestor between the sequences, whereas selection on long-separated, independently evolving lineages would act only on non-synonymous changes and would likely affect only a subset of those sites. Horizontal transfer can occur via vectors, such as bacteria, fungi, or sap-sucking insects [61–63], or through hybridization and introgression (transfer of genetic material from the genome of one species to another via recurrent backcrossing of a hybrid with one of its parents). The transfer of TEs via hybridization may seem at first less likely to explain the observed relationships among these *PIF*-elements, because it requires hybridization among genera. However, the evolutionary history of the wheat tribe has been extensively studied, and Triticeae grasses provide a particularly striking example of a group in which reticulations are common [32, 34, 64]. Polyploids have long been suspected to serve as genetic bridges between diploid species [64], and if introgression is responsible for the sporadic distribution of these *PIF* transposases, it might proceed via polyploids.

There is no straightforward way to distinguish whether our results reflect vector-mediated HT or hybridization without examination of the flanking regions of the TE and the identification of a common vector. However, phylogenetic analyses of Triticeae based on multiple genes trees provide evidence that hybridization and introgression do play a role in the wheat tribe's history. For example, chloroplast DNA (cpDNA) data have grouped *Pseudoroegneria*, *Thinopyrum*, and *Dasypyrum* in a well-defined and mostly well-supported clade [34], whereas these three genera are not closely related in comparably-sampled nuclear gene trees(e.g., [31, 32, 51]).This suggests that past introgressive events have played a significant role in shaping Triticeae genomes, and that introgression is a feasible explanation for the presence of nearly-identical elements shared by these same three genera (*D*. *villosum* 10; *P*. *libanotica* 5 and 14; and *T*. *bessarabicum* 8 and 13). Despite the vast number of *PIF* elements in plants, this is the first convincing evidence of HT of *PIF*-like elements in plant genomes.

Although we can not generalize our results to other DNA transposons, we have obtained similar results in our investigation of Pong elements (http://dx.doi.org/10.1016/j.ympev.2015.07.008), another family of transposons in the *PIF*/Harbinger superfamily. Both *PIF* and Pong TEs show similar evolutionary trajectories within the wheat tribe, best explained by descent from diverse ancestral transposases and occasional HT events between genera. Compared to Pong, the expansion of *PIF* elements within Triticeae seems to be more recent. Most *PIF* sequences appear to be relatively young, with a maximum sequence divergence of 27.5%, compared to a maximum of 52.35% divergence for Pong elements from the same host plants. However, it is also possible that we have amplified only a subset of existing *PIF* elements, or that they evolve more slowly.

### Detecting signatures of selection in 126 *PIF*-like transposase sequences

After their propagation within a genome, TEs may lose their function, and rapidly accumulate mutations following a neutral model of evolution akin to pseudogenes [65–67]. In contrast, elements that remain functional are expected to evolve either under purifying or positive selection. Many of the elements in our sample have acquired frame-shifting or nonsense mutations, and are thus probably non-functional. However, among the sequences lacking frame shifting indels or stop codons, apart from few artifactual *d*N/*d*S, our results revealed a dominant signal of negative selection (average values of *d*N/*d*S of 0.2026). This value is similar to those calculated for nuclear genes evolving under strong selective constraints [68], indicating that the transposase is under strong purifying selection in Triticeae (or it was in the recent past). Purifying selection was also detected for *PIF* sequences in the genome of most Drosophila lineages [19]. Our *d*N/*d*S values for these 126 transposase fragments suggest recent *PIF* activity in Triticeae, such as transposition or mobilization of related non-autonomous elements. A phylogenetic analysis of these fragments (Fig 2) also showed that they are derived from multiple evolutionary lineages, indicating that multiple transposases can function simultaneously within a genome.

## Conclusion

Our detailed analyses of *PIF*-like transposase paralogues in Triticeae genomes showed that *PIF* elements are widely dispersed within the wheat tribe, and have been maintained by vertical transfer with stochastic loss and horizontal transfer between genera. We identified at least four main groups of *PIF* transposases, with some that appeared to be currently or recently active. Their evolutionary history is complex, and our proposed explanations (ancestral polymorphism, stochastic loss and retention of *PIF* copies, different evolutionary success, vector-mediated introgressive events, and/or past and ongoing hybridization events) are not mutually exclusive. More exhaustive analysis of element transcription and activity is necessary to assess the impact of *PIF*-like elements on host genomes.

## Supporting Information

S1 Table Nonsynonymous substitutions of 126 *PIF*-like transposase fragments. (XLS)

S2 Table Synonymous substitutions of 126 *PIF*-like transposase fragments. (XLS)

S3 Table Selection test: neutrality of 126 *PIF*-like transposase fragments. (XLS)
